# Metagenome-assembled genome sequences of two bacterial species from polyvinyl alcohol-degrading co-colonies

**DOI:** 10.1128/mra.00363-24

**Published:** 2024-06-11

**Authors:** Kohei Nakamura, Sota Asano, Masashi Nambu, Nanami Ishiguro, Atsushi Tanikawa, Nobuaki Naganuma

**Affiliations:** 1 Faculty of Applied Biological Sciences, Gifu University, Gifu, Japan; 2 Basic Technology Research Division R&D Department, AICELLO CORPORATION, Aichi, Japan; Indiana University, Bloomington, Bloomington, Indiana, USA

**Keywords:** pollutant, polymers, polyvinyl alcohol, symbiosis

## Abstract

We present the metagenome-assembled genome sequences of two polyvinyl alcohol-degrading co-colony-derived bacterial species relative to *Rhodanobacter* sp. DHB23 and *Priestia megaterium* ATCC 14581. We estimated the genomes of these species to be 3,476,996- and 5,169,587-bp long (for *Rhodanobacter* sp. DHB23 and *Priestia megaterium* ATCC 14581, respectively).

## ANNOUNCEMENT

Various industries extensively use polyvinyl alcohol (PVA) although it pollutes the environment (1). Even with activated sludges (AS) in municipal wastewater treatment plants (MWWTP), decomposing PVA is difficult (2, 3). Moreover, PVA-degrading microorganism genomes and ecology in environments like AS are poorly understood. Here, we describe MWWTP AS-derived PVA-degrading species and their genome sequences.

We obtained an AS sample from the MWWTP of Toyohashi City, Japan. We inoculated the AS washed with an inorganic medium (4) into 50 mL of the inorganic medium, amended with 0.02% PVA [SOLUBLON GA (Mn: 44000), Aicello CO LTD, Japan] in a 500-mL shaking flask and incubated at 60 rpm and 30°C. We measured the PVA concentration within the culture supernatant using the Deng method (5) and spread the culture dilutions onto a 0.02% PVA-containing agar plate, cultivated at 30°C to screen PVA-degrading colonies. We repeatedly streaked a colony, named ACL01, forming a transparent circle on an iodine-stained PVA agar plate, for purification.

We grew ACL01 cells on the PVA agar at 30°C for 4 days, harvested by centrifugation, and extracted their DNA using the DNeasy Blood & Tissue Kit (QIAGEN), following the manufacturer’s protocol for Gram-positive bacteria. The extracted DNA was used for following sequencings without shearing and size selection. We performed short-read sequencing using the Illumina MiSeq platform with a v3 reagent kit and 2 × 300 bp paired-end reads, along with the Illumina DNA Prep kit and IDT for Illumina Nextera DNA CD Indexes. Long-read sequencing was performed using the Oxford Nanopore MinION platform with the Rapid Barcoding kit and R9.4.1 flow cell. We obtained 1,048,348 and 556,568 reads from MiSeq and MinION, respectively.

We used the default parameters for all software processing unless stated otherwise. We trimmed raw sequencing reads for quality and sequencing adapters using fastp v0.21.0 (6) for MiSeq and Guppy basecaller v6.3.9 with a superaccurate base-calling model, and Porechop (v.0.2.4 https://github.com/rrwick/Porechop) for MinION. We performed a hybrid assembly using filtered reads with MUFFIN (7) with modifications (--assembler metaflye—filtlong --modular assemble). Our results yielded four closed 3,476,996-, 5,169,587-, 19,104-, 52,234 bp contigs. Circularities of the contigs were confirmed by visualizing *de novo* assembled graph depicted with Bandage v.0.8.1 (8). We computed the statistics of the assembled contigs using QUAST v.5.0.2 (9) (Table 1), rotated, annotated, and classified the sequences using Prodigal v.2.6.3 (10), ARAGORN v.1.2.38 (11), CRT v.1.2 (12), barrnap v.0.8 (13), CheckM (14), and GTDB-Tk (15) in the online DFAST (16). DDBJ/ENA/GenBank accession numbers of sequences are shown in Table 1.

**TABLE 1:** Sequencing metrics and genome statistics

	Sequence1		Sequence2	Sequence3		Sequence4	
Raw sequence reads							
Illumina paired-end read length (nucleotides)	300						
Number of Illumina reads used	1,048,348						
Number of Nanopore reads used	556,568						
Nanopore read *N*50 (bp)	9,471						
Assembly statistics for closed sequences^*a*^							
Total sequence length (bp)	3,476,996		5,169,587	19,104		52,234	
Number of sequences	1		1	1		1	
GC content (%)	67.3		38.2	35.3		35.3	
Number of CDSs	3,077		5,217	0		67	
Average protein length	340.9		275.3	NA*^**b**^*		213.7	
Coding ratio (%)	90.5		83.3	NA		82.3	
Number of rRNAs	6		40	0		0	
Number of tRNAs	57		115	0		2	
Average Illumina coverage (×)	6		34	88		54	
Mapped Illumina reads (%)	11.66		77.92	0.74		1.29	
Average Nanopore coverage (×)	210		366	284		1,500	
Mapped Nanopore reads (%)	24.62		73.32	0.28		1.78	
CheckM result							
Completeness, contamination, strain heterogeneity (%)	100, 0.09, 0		99.35, 0.32, 0	NA		NA	
Taxonomy assignment							
GTDB Taxonomy (accession no., ANI^*c*^ %)	*Rhodanobacter* sp. (GCF_014842975.1, 89.01)		*Priestia megaterium* (GCF_000832985.1, 96.24)	NA		NA	
GenBank accession no.							
BioProject accession no.	PRJDB17550						
Sequence Read Archive no.	DRR538500 (Nanopore)			DRR538501 (Illumina)			
BioSample accession no.	SAMD00760402		SAMD00760403	SAMD00760404		SAMD00760405	
Genome assembly accession no.	AP031382		AP031383	LC810154		LC810155	

^*a*^ No CRISPR arrays were detected in all sequences.

^*b*^ Not applicable.

^*c*^ Average nucleotide identities.

Two bacterial genomes were retrieved from ACL01 colonies on the PVA plate which were morphologically uniform in shape. MAG sequences 1 and 2 were related to *Rhodanobacter* sp. DHB23 and *Priestia megaterium* ATCC 14581 (ANI values: 89.04% and 96.24%), respectively. The origins of sequences 3 and 4 remain clear. We found a homolog of PVA dehydrogenase in sequence 1 (Genbank: BFI96117.1) and oxidized PVA hydrolase homologs in either sequence 1 (Genbank: BFI95560.1) or sequence 2 (Genbank: BFI99320.1 and BFJ00521.1). The two species form co-colonies on the PVA agar and may collaborate to decompose PVA using those enzymes. The MAG sequences could advance studying the associations between the co-colony-forming species and provide significant insights into PVA-degrading microbes in various environments.
